# Time-Controlled Adaptive Ventilation (TCAV): a personalized strategy for lung protection

**DOI:** 10.1186/s12931-023-02615-y

**Published:** 2024-01-18

**Authors:** Hassan Al-Khalisy, Gary F. Nieman, Michaela Kollisch-Singule, Penny Andrews, Luigi Camporota, Joseph Shiber, Toni Manougian, Joshua Satalin, Sarah Blair, Auyon Ghosh, Jacob Herrmann, David W. Kaczka, Donald P. Gaver, Jason H. T. Bates, Nader M. Habashi

**Affiliations:** 1 East Carolina University, Greenville, NC USA; 2 SUNY Upstate Medical University, 750 E. Adams St., Syracuse, NY 13210 USA; 3 R Adams Cowley Shock Trauma Center, University of Maryland Medical Center, Baltimore, MD USA; 4 Health Centre for Human and Applied Physiological Sciences, Guy’s and St Thomas’ NHS Foundation Trust, London, UK; 5 University of Florida College of Medicine, Jacksonville, FL USA; 6 Westchester Medical Center, Valhalla, NY USA; 7 University of Iowa, Iowa City, IA USA; 8 Tulane University, New Orleans, LA USA; 9 University of Vermont, Burlington, VT USA

**Keywords:** Acute respiratory distress syndrome, Ventilator-induced lung injury, Open lung approach, Dynamic alveolar mechanics, Regional alveolar instability, Viscoelastic, Stress-multipliers, Alveolar opening and collapse time constants, Tidal volume, Driving pressure, ARMA, APRV, TCAV, VILI, ARDS

## Abstract

Acute respiratory distress syndrome (ARDS) alters the dynamics of lung inflation during mechanical ventilation. Repetitive alveolar collapse and expansion (RACE) predisposes the lung to ventilator-induced lung injury (VILI). Two broad approaches are currently used to minimize VILI: (1) low tidal volume (LV_T_) with low-moderate positive end-expiratory pressure (PEEP); and (2) open lung approach (OLA). The LV_T_ approach attempts to protect already open lung tissue from overdistension, while simultaneously resting collapsed tissue by excluding it from the cycle of mechanical ventilation. By contrast, the OLA attempts to reinflate potentially recruitable lung, usually over a period of seconds to minutes using higher PEEP used to prevent progressive loss of end-expiratory lung volume (EELV) and RACE. However, even with these protective strategies, clinical studies have shown that ARDS-related mortality remains unacceptably high with a scarcity of effective interventions over the last two decades. One of the main limitations these varied interventions demonstrate to benefit is the observed clinical and pathologic heterogeneity in ARDS. We have developed an alternative ventilation strategy known as the Time Controlled Adaptive Ventilation (TCAV) method of applying the Airway Pressure Release Ventilation (APRV) mode, which takes advantage of the heterogeneous time- and pressure-dependent collapse and reopening of lung units. The TCAV method is a closed-loop system where the expiratory duration personalizes V_T_ and EELV. Personalization of TCAV is informed and tuned with changes in respiratory system compliance (C_RS_) measured by the slope of the expiratory flow curve during passive exhalation. Two potentially beneficial features of TCAV are: (i) the expiratory duration is *personalized* to a given patient’s lung physiology, which promotes alveolar stabilization by halting the progressive collapse of alveoli, thereby minimizing the time for the reopened lung to collapse again in the next expiration, and (ii) an extended inspiratory phase at a fixed inflation pressure after alveolar stabilization gradually reopens a small amount of tissue with each breath. Subsequently, densely collapsed regions are slowly ratcheted open over a period of hours, or even days. Thus, TCAV has the potential to minimize VILI, reducing ARDS-related morbidity and mortality.

**Graphical Abstract:**

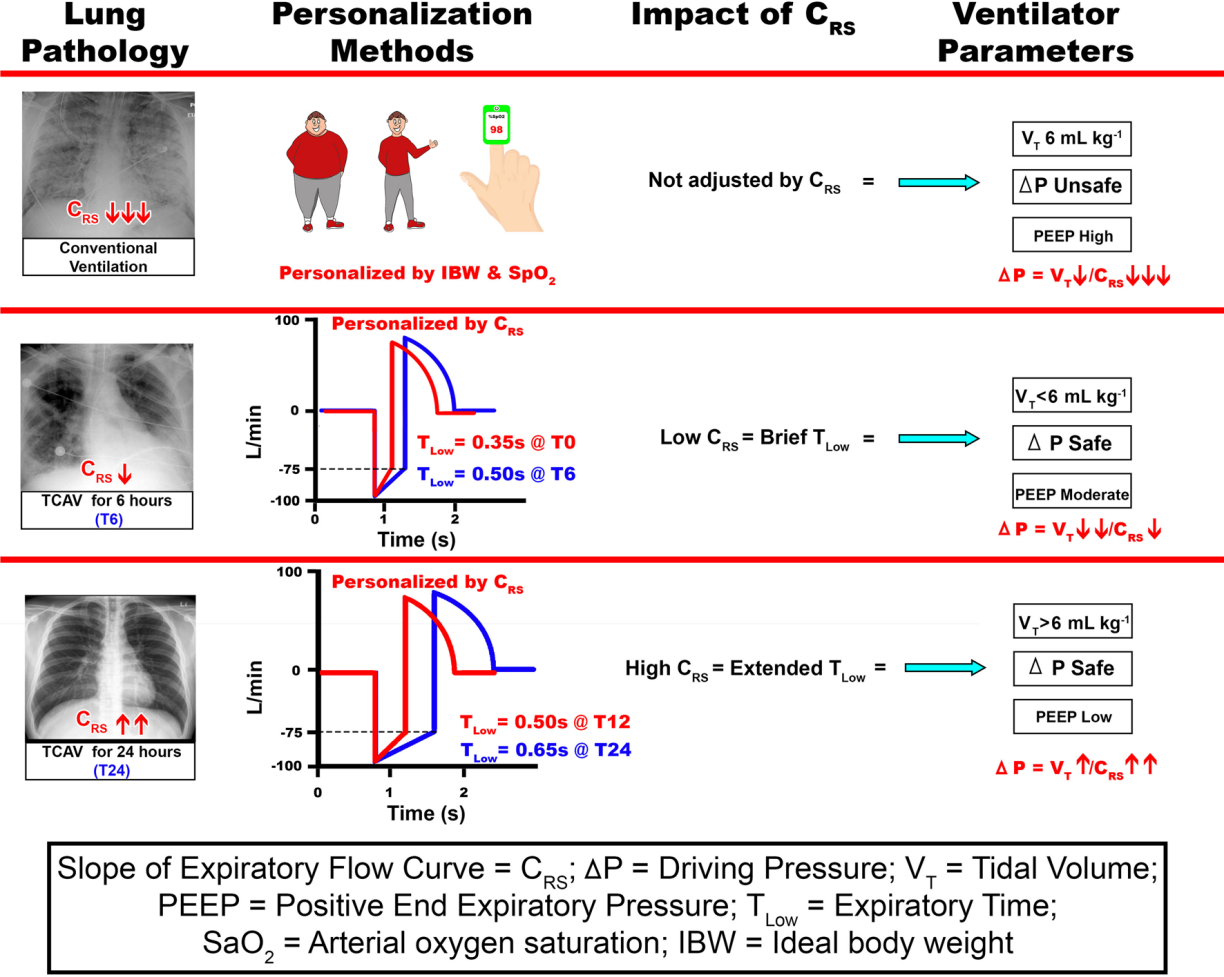

**Supplementary Information:**

The online version contains supplementary material available at 10.1186/s12931-023-02615-y.

## Introduction

Acute respiratory distress syndrome (ARDS) remains a significant clinical problem, with primary management being supportive mechanical ventilation [1]. However, mechanical ventilation itself has the potential to be damaging by causing ventilator-induced lung injury (VILI), which significantly increases ARDS-related mortality [2]. Current “protective” ventilation strategies are aimed at reducing VILI; however recent studies indicate that current lung protective ventilation strategies have not reduced ARDS-associated mortality [3–6]. The reasons for this remain unclear, but there are obvious limitations to mechanical ventilation in ARDS based on clinical trials addressing “one-size-fits-all” approaches applied to a heterogeneous patient population. For example, the use of a tidal volume (V_T_) of 6 mL kg^−1^ of ideal body weight (IBW) might be safer than 12 mL kg^1^ on average, although it is highly unlikely that 6 mL kg^−1^ is optimal for any given patient. Thus, there is an urgent need to find alternative, personalized approaches to mechanically ventilating the injured lung that takes individual patient pathophysiology into account. Devising such a personalized approach to ventilation starts with an understanding of the underlying pathophysiology of ARDS.

A breach of the blood-gas barrier in ARDS allows protein-rich fluid to accumulate in the distal airspaces of the lung, where it inactivates pulmonary surfactant. This surfactant dysfunction increases surface tension at the air–liquid interface, with major consequences for alveolar and acinar inflation [7–11]. An early [and still prevailing] concept is that the lung becomes separated into two functionally distinct compartments along the gravitational axis. In gravitationally dependent regions where edema fluid accumulates and the most severe surfactant dysfunction occurs, alveoli and small airways collapse and/or become filled with fluid, to form atelectatic regions that do not participate in gas exchange. In non-dependent regions, which are largely devoid of edema, the parenchyma remains essentially normal and can be ventilated. This ventilated compartment, however, is reduced in volume compared to the whole lung, thus being referred to as the “baby lung” [12]. More recent experimental work, informed by imaging with paired high-resolution computerized tomography (CT) and Helium magnetic resonance imaging provide sub-acinar resolution in human and animal studies, has made it clear that the situation is more complex than implied by this simple binary compartmentalization [10, 13–16]. In particular, tissues at the interface between open and cyclically closed regions are particularly susceptible as mechanisms of VILI [overdistension and *repetitive alveolar collapse and expansion* (RACE)] amplify and propagate due to anisotropic distortions [17]. This damaging RACE during mechanical ventilation are phenomena that depend on both *time* as well as *pressure* [18, 19], and should be considered when designing protective and personalized ventilation strategies.

In this review, we will examine the mechanistic underpinnings of conventional ventilation strategies for ARDS that are presumed to be protective. We then describe an alternative, but promising, strategy that has the potential to be both protective and personalized, by accounting for the time and pressure dependencies of RACE.

### Conventional protective ventilation strategies

#### Low tidal volume approach

The original ARDS Network (ARDSNet) low tidal volume (LV_T_) approach (i.e., 6 mL kg^−1^ of IBW) is to protect the normal lung from overdistension injury (volutrauma). Persistently collapsed lung tissue is allowed to ‘rest’ by remaining unventilated [12, 20, 21]. This LV_T_ approach also strives to keep plateau airway pressure (P_plat_) less than 30 cmH_2_O with the application of positive end-expiratory pressure (PEEP) guided by oxygenation necessary to prevent progressive loss of end-expiratory lung volume (EELV) and minimize RACE-induced atelectrauma [20, 22].

Currently, the LV_T_ approach is the standard of care for patients with ARDS. However, ARDS-related mortality remains unacceptably high and has shown little or no improvement with LV_T_ [3–6]. For example, the 2,587 patients that were eligible but not enrolled for technical reasons from the 2000 ARMA trial, [but nevertheless treated with the prior standard of care V_T_ of approximately 10 mL kg^−1^] [23] were analyzed by Deans et al. [24] and found to have the same mortality as the LV_T_ group. Also, V_T_ of 12 mL kg^−1^ was not always associated with increased mortality, nor was LV_T_ always associated with lower mortality. Rather, raising V_T_ increased mortality (42% vs. 29%) in patients with low respiratory system compliance (C_RS_), but reduced mortality (21% vs. 37%) in those with higher C_RS_ [24]. So, lung protection or injury is not dictated solely by the size of V_T_. It also depends on the extent of inflatable tissue, indicated by C_RS_, that receives V_T_, along with the seriousness of lung pathophysiology [25]. A more recent study reviewing ARDS mortality in 18 intensive care units (ICUs) showed that patients receiving V_T_ of 4–6 mL kg^−1^ had a higher rate of mortality than those receiving 6–10 mL kg.^−1^[26].

More recently, it has been shown that driving pressure (ΔP) and mechanical power (MP) are better surrogates for VILI than the size of the V_T_ [27]. To maintain ΔP and MP within the safe range, adjustments are made at the ventilator using lower V_T_ and reducing peak (Ppeak) and plateau airway pressures. By simply ‘treating the ventilator’ (changes V_T_ and airway pressures) the physician is constrained to ventilating a collapsed, heterogeneously injured lung. A better idea would be to fully reopen the lung, removing the constraints of ventilating a heterogeneously injured lung, and lower ΔP by increasing C_RS_, since ΔP = V_T_/C_RS_. An approach to accomplish this goal will be discussed in the “*A Personalized Approach to Mechanical Ventilation”* section below.

**Fig. 1:**
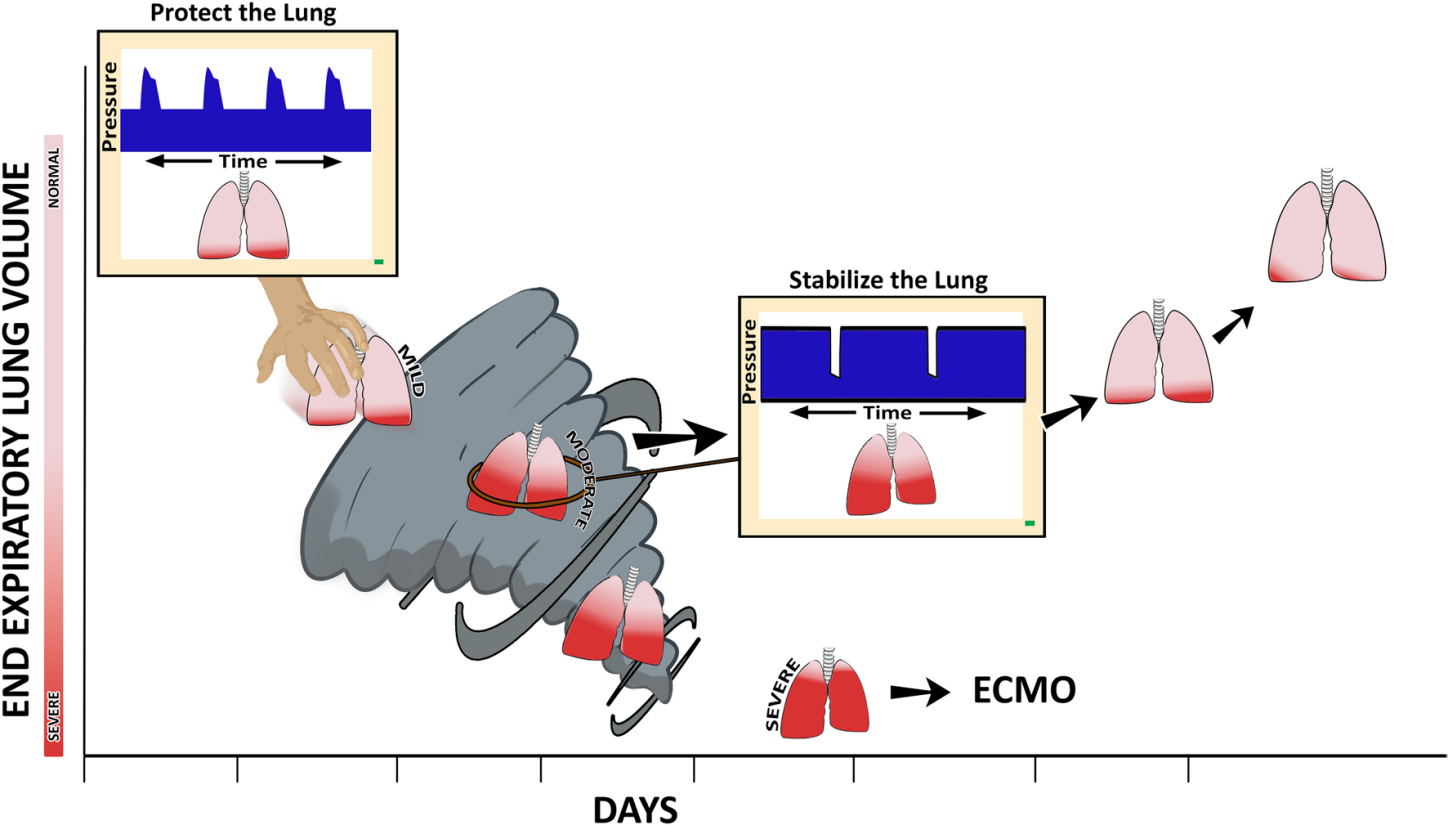
An ever-shrinking, baby lung, known as a VILI Vortex has been used to describe the evolution of ventilator-induced lung injury (VILI) [28]. Upper left: The ‘patient’ with mostly open lung tissue (pink) and a lesser amount of collapsed tissue (red) defined as Mild ARDS is placed on ARDSnet LV_T_ ventilation. The LV_T_ and low airway pressures strategy is designed to ‘rest’ the ‘baby lung’, however, this ventilation method allows the acutely injured tissue to continually collapse pushing it into the VILI Vortex. Lung pathogenesis moves from Mild to Moderate ARDS as normal tissue progressively shrinks (pink → red), Disease progression into Severe ARDS is inevitable if unchecked at which point rescue methods such as extracorporeal membrane oxygenation (ECMO) may be necessary. ARDS causes the lung to become *time* and *pressure* dependent. This means that it will take more time for the alveoli to open and less time for them to collapse at any given airway pressure. Thus, the alveolar opening can be accelerated by an extended inspiratory time, and alveolar collapse minimized by a short expiratory time. The brief *time* at inspiration is not adequate to open collapsed alveoli while the extended *time* at expiration will not prevent alveolar collapse using the ARDSnet approach (Upper left Protect the Lung, Ventilator Monitor blue Pressure/Time curve). The open lung approach to rapidly reopen the lung (seconds or minutes) using recruitment maneuvers and higher PEEP has not been successful at reducing ARDS-related mortality. Using inspiratory and expiratory duration in addition to pressure to open and stabilize alveoli has been shown very effective and lung protective by our group and others [19, 72, 75–91]. An extended inspiratory time will progressively recruit alveoli and a brief expiratory time will prevent re-collapse. A correctly set time-controlled ventilator method will stabilize alveoli (Center, Stabilize the Lung, Ventilator Monitor blue Pressure/Time curve) using a short expiratory time pulling the lung from the Vortex. Once progressive lung collapse is halted, collapsed tissue can be reopened slowly (Red lung tissue turning pink) over hours or days depending on the level of lung pathophysiology [19, 72, 75–91]. This figure depicts the ability of TCAV to be used after ARDS has developed or as a rescue mode but if applied early during mild ARDS movement of the lung into the Vortex could be prevented. Reproduced from Reference [29], under terms of the Creative Commons Attribution 4.0 International License

The lack of meaningful ARDS mortality reduction with LV_T_ can be potentially explained by several factors. One factor is the application of PEEP does not prevent the gradual de-recruitment of lung regions due to the transience of the open lung compartment. Lung recruitment may initially occur following the application of high airway pressure, such as during a sigh breath; however, lung collapse can gradually recur over time. This led Marini and Gattinoni to describe the “VILI Vortex” in which continued shrinking of the baby lung places increasing strain on the remaining aerated tissue [28, 29], leading eventually to the need for rescue strategies such as high-frequency ventilation (HFOV) or extracorporeal membrane oxygenation (Fig. 1). Further, overdistension of the baby lung may not be the primary mechanism driving VILI, because normal lung tissue is highly resistant to injurious overdistension [30–39]. By contrast, overdistension, and diffuse micro-atelectasis are clustered in adjacent lung regions and highly damaging in the presence of RACE [22], not only to the unstable or collapsed alveoli but also to the adjacent alveoli that share alveolar walls (Additional file 1, Additional file 2) [8–10, 17, 39, 40], These phenomena cannot be observed on chest radiograph or standard CT imaging with a conventional breath hold [11].

The presence of persistent collapsed lung tissue can lead to other pathologies or pathophysiologic processes, such as fibrosing alveolitis [41, 42], increased pulmonary vascular resistance (PVR), patient/ventilator asynchrony, surfactant inactivation, right ventricular strain, and/or right ventricular failure [43]. Although ‘resting’ the collapsed lung sounds protective, long-term atelectasis generates multiple pathophysiologic problems. Ventilation/perfusion surface area shrinks with atelectasis leading to hypoxemia and hypercapnia, thus increasing FiO_2_ requirements and the subsequent risks of oxygen toxicity and absorption atelectasis and fibrosis [41, 44]. Progressive reduction of EELV puts more stress and strain on the remaining normal tissue lung tissue that will now receive all of the V_T_ driving the lung into the ‘VILI Vortex’ (Fig. 1) [28]. Loss of EELV will increase PVR [45, 46] which may require vasoactive agents that may not prevent progression to right heart failure [47]. Long-term lung collapse is known as collapse induration and fibrosing alveolitis resulting in permanent dysfunctional and fibrotic tissue [42, 44, 48, 49]. Atelectasis is independently associated with loss of surfactant function and lung stretch. Surfactant is necessary to facilitate the expansion of collapsed lung and requires a gas interface and cyclic stretching to stimulate type 2 pneumatocytes cell to produce surfactant B. During mechanical ventilation, atelectatic regions need gas and cyclic stretching to stimulate exogenous surfactant release [50, 51]. Hypoxemia, hypercapnia, and stretch receptors in the atelectatic tissue cause dyspnea resulting in patient-ventilator asynchrony [52], and finally collapsed lung tissue increases the risk of developing pneumonia [53, 54].

#### Open lung approach

As described by Marini and Gattinoni, the VILI Vortex results in a shrinking of the baby lung, such that normal lung tissue is progressively lost to ongoing collapse (Fig. 1) [28, 29]. This results in increased strain on the remaining aerated lung, presumably increasing the risk of VILI. The Open Lung Approach (OLA) attempts to avert this situation, by applying sufficient PEEP, with or without periodic recruitment maneuvers (RMs), such that progressive derecruitment and loss of EELV will be minimized [55–58]. Unfortunately, the mortality associated with ARDS rates has not been reduced with the use of OLA [55–58], relative to that in the original ARMA study [20], with an increase in mortality in the OLA group (55.3% vs 49.3%) in the recent Alveolar Recruitment for Acute Respiratory Distress Syndrome Trial [55].

HFOV, which can achieve a higher level of mean airway pressure (Paw) than conventional modes, can also be considered an OLA strategy but has not been shown to improve ARDS mortality compared to conventional lung protective ventilation [59–61]. For example, in the Oscillate trial, following a RM of 40 cmH_2_O airway pressure for 40 s, the Paw was set from 20 to 38 cmH_2_O based on oxygenation [59]. The lack of efficacy may be due to the fact that HFOV flow may be heterogeneously distributed throughout the lung [62], leading to wide variations in parenchymal strain and potential for worsening injury [13].

Given the physiologic rationale for eliminating collapsed lung tissue, negative results from clinical trials incorporating OLA are surprising. Perhaps assuming that simply opening the injured lung will protect from further VILI [59–61] has revealed gaps in our understanding. Data from a recent OLA trial showed no clear evidence of long-term recruitment. These data included: (1) low C_RS_ in the OLA group that was not significantly different from the control group at Day-7 (34.5 vs 32.4 mL cmH_2_O^−1^) and (2) PaO_2_/FiO_2_ ratios [although significantly higher in the OLA than the control group] remained in the mild ARDS category at Day-7 (262.7 vs 215.1) [55]. In addition, patients that tolerate OLA strategies typically require deep sedation with use of neuromuscular blocking agents which demonstrate inconsistent benefit and potential to worsen outcomes [63, 64]. The failures of OLA have led some to call for its abandonment in the clinical management of ARDS, even though the likely reason for such failures is that the expected level of lung recruitment was not achieved [65]. This is counterintuitive, because the scientific approach can be used to for an evidence-based solution. Given the evidence that atelectrauma caused by RACE and the volutrauma caused by micro-atelectasis are key mechanisms driving VILI, further clarity is needed [11, 17, 30, 33, 34, 40, 66]. Thus, abandoning OLA may close the door on a lung protective approach that could ultimately be successful, if applied in a clinically appropriate manner.

Unfortunately, the rate at which the lung is reopened remains in question and current methods attempt to force open the lung in seconds [67] or minutes [55]. Recruiting the acutely injured lung with dysfunctional surfactant quickly is problematic since the current OLA strategies only use increased airway pressure in the form of PEEP to prevent re-collapse. A large volume of open lung tissue without functional surfactant would require a very high PEEP to prevent de-recruitment. Perhaps a better method to achieve goals of the OLA is to encourage recruitment gradually, recognizing the lung’s time dependencies [68]. Indeed, the optimal method to reopen the collapsed lung may take hours or even days depending on the severity of lung injury.

Recent studies have identified ARDS phenotypes that affect disease pathogenesis and outcomes [69]. We postulate that phenotypes have more of an impact on the disease (ARDS due to either systemic or local inflammation) than on the secondary injury (VILI caused by mechanical damage) [69]. There is little literature on patient phenotype modifying the impact of VILI, but in vitro studies suggest the possibility [70]. The ARDS patient phenotype may be important in VILI if atelectrauma and volutrauma exacerbate the inflammatory cell response (biotrauma). However, biotrauma would not be an issue if protective mechanical ventilation can minimize atelectrauma and volutrauma.

### A personalized approach to mechanical ventilation

The aforementioned problems with LV_T_ and OLA suggest improved outcomes in ARDS might be achieved with a ventilation strategy that is able to reverse progressive alveolar instability to stop RACE and subsequently deliberately open densely collapsed lung and keep it in that state for the duration of the patient’s course in the ICU [71]. This would effectively halt the VILI Vortex, by eliminating the nidus of both atelectrauma and volutrauma (Fig. 1) [29, 72]. The question is how to achieve this outcome. Forcibly reopening collapsed atelectatic parenchyma typically takes significant pressure. For example, in the ART trial, recruitment was a 3-step process with step 3 being a peak recruiting pressure of 50cmH_2_O [55]. Thus, it is easy to imagine that over time, the process of recurring and unnatural rapid reopening of the lung with current RM strategies results in more tissue damage than if the lung were allowed to remain collapsed. We propose the answer to this conundrum lies in controlling the rate at which the atelectatic lung is reopened. Specifically, the goal should be to achieve the normally opened state slowly over extended periods of time, such that only incremental opening, and thus minimal tissue damage, occurs with each breath (Additional file 3, Additional file 4). In support of this idea, Dianti et al. [73] found ARDS mortality decreased with higher levels of PEEP and occasional RMs.

It is also crucial to avoid RACE with each expiration as rapidly as possible since this would eliminate atelectrauma, a key and proximal VILI mechanism. Since alveoli, are interconnected, polygonal, and share walls, atelectrauma propagates and predisposes the lung to additional volutrauma, a key consequential VILI mechanism, by overdistending the adjacent open alveoli when they collapse during exhalation [30–32, 38, 39]. If the opening or closing of a particular unit depended only on the pressure applied to it, the most logical approach would be to maintain airway pressure above the highest closing pressure *at all times*. Raising PEEP to the necessary level in ARDS, however, is often untenable, because closing capacity and the pressures to maintain the lung open can be substantially elevated and divergent in the injured lung. Moreover, conventional ventilation still requires sufficient driving pressures above PEEP to maintain adequate ventilation. Perhaps underappreciated, the opening and closing of small airways and alveoli also depend on time. That is, a unit does not close immediately when exposed to its required closing pressure. Rather, there is a delay before closure occurs.

The fluid that lines the airspaces must flow to the point of closure, which takes time depending on the volume and viscosity of the fluid, as well as the surface tension at the interface. Moreover, changes in airway pressure are not transmitted immediately to the distal lung regions, due to the resistive pressure drop across the intervening conducting airways. Accordingly, changes in airway pressure should not be used to infer corresponding changes in alveolar volume [40, 66, 74]. This affords the opportunity to avoid closure by keeping the duration of expiration less than the closing delay. The question is whether it also allows enough time to achieve the minute ventilation necessary for gas exchange, particularly since the lung requires more time to open (and less time to collapse) as its injury worsens. Thus, an appropriate balance between these two competing processes needs to occur.

#### Stabilize and gradually recruit approach

**Fig. 2:**
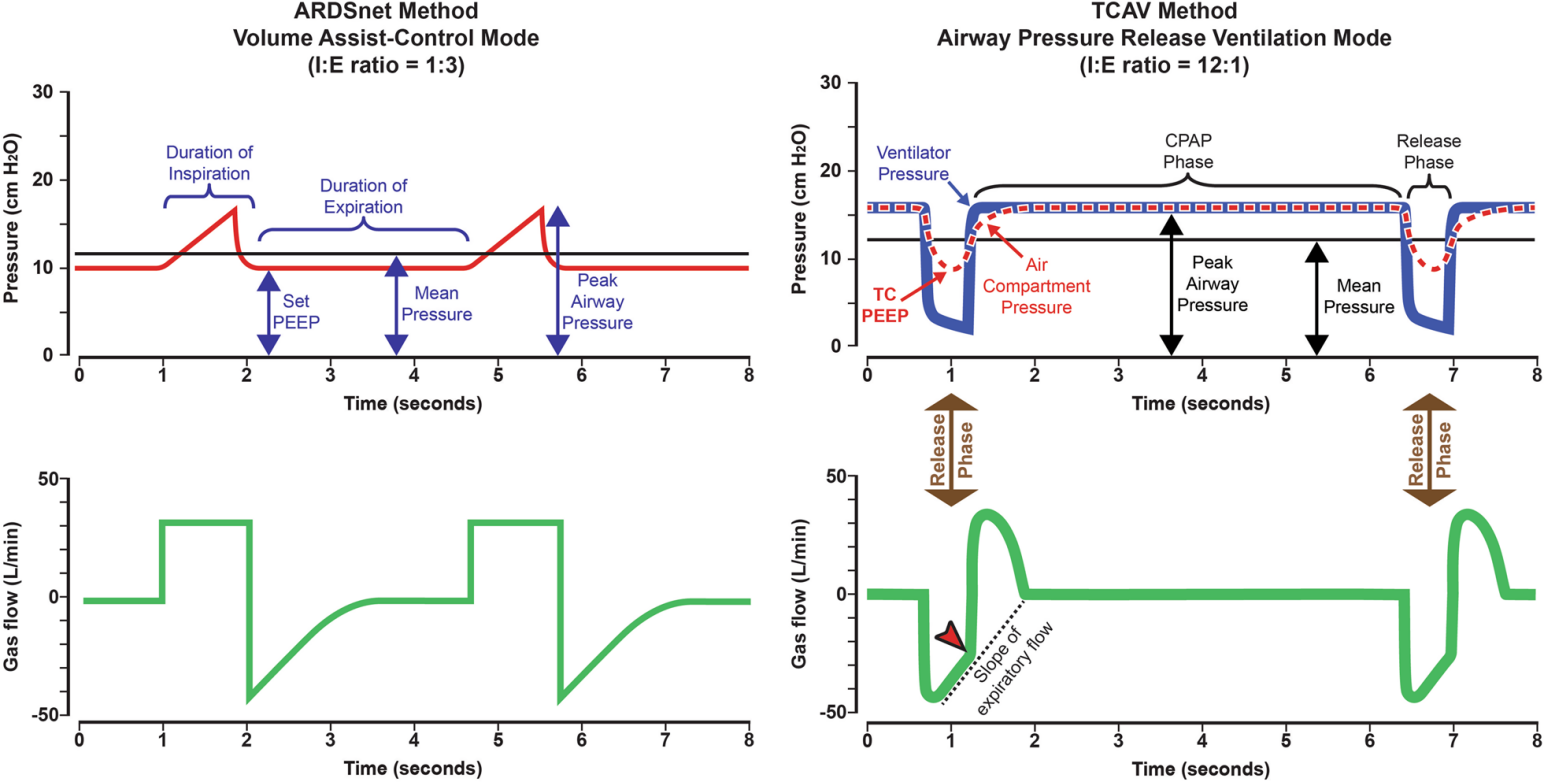
**A** Pressure/Time and Flow/Time curves = generated by the ARDSnet method to set and adjust the Volume Assist-Control mode. Key features include an inspiratory: expiratory ratio of 1:3 where the peak/plateau inspiratory pressure is brief. A set positive end-expiratory pressure (Set-PEEP) and FiO_2_ are adjusted using oxygenation as the trigger for change [20]. **B** Pressure/Time and Flow/Time curves for the Time Controlled Adaptive Ventilation (TCAV) method to set and adjust the Airway Pressure Release Ventilation (APRV) mode. Key features include an inspiratory: expiratory ratio of ~ 12:1, where. the continuous positive airway pressure (CPAP) Phase is ~ 90% of each breath. A tidal volume (V_T_), which is measured as the volume of gas released during the Release Phase (brown arrow)**,** is not set but is influenced by changes in (i) respiratory system compliance (C_RS_), (ii) the CPAP Phase pressure, and (iii) the duration of the Release Phase. The Release Phase is determined by the Slope of the Expiratory Flow Curve (red arrowhead), which is a breath-to-breath measure of C_RS_. The lower the C_RS,_ the faster the lung recoil, the steeper the slope, and the shorter the Release Phase, further reducing V_T_. Thus, the V_T_ will be low in a non-compliant, injured lung and will increase in size *only* when the lung recruits and C_RS_ increases. Since a change in C_RS_ directs the Release Phase duration, which in turn adjusts the V_T_ and the time-controlled PEEP (TC-PEEP) the TCAV method is both *personalized* and *adaptive* as the patient’s lung gets better or worse [104]. Reproduced from Reference [104], under terms of the Creative Commons Attribution 4.0 International License

One adaptive ventilation strategy that avoids closure during each expiration while ratcheting open densely collapsed atelectatic lung is the Time Controlled Adaptive Ventilation (TCAV) method [19, 33, 72, 75–91] to set and adjust the Airway Pressure Release Ventilation (APRV) mode. Although the APRV mode is often considered another OLA, this is not a realistic comparison when using the TCAV method. The OLA (40 cmH_2_O airway pressure for 40 s) attempts to open most of the collapsed lung within seconds or minutes [92]. The immediate effect of the TCAV approach, on the other hand, is to stabilize the lung using a short duration of expiration that does not allow the alveoli enough time to de-recruit, effectively establishing a time-controlled PEEP (TC-PEEP, Fig. 2, TCAV Method Release Phase). The subsequent re-opening of de-recruited lung may be gradual, taking possibly hours or even days, which is less damaging to injured tissue than forcing it to open quickly.

Using TCAV, an upper pressure (P_High_) is applied for an extended duration (T_high_) that facilitates gradual reopening and maintains patent lung units inflated throughout most of the breath cycle referred to as the continuous positive airway pressure (CPAP) Phase. The preceding brief time (T_Low_) spent at the lower pressure (P_Low_) known as the Release Phase has halted expiratory airway closure so any inspiratory airway reopening remains durable. The combined diffusive (CPAP phase) and convective (release phase) gas exchange provides efficient CO_2_ removal. The following will discuss the physiologic impact of using inspiratory and expiratory time to stabilize and reopen the lung. Detailed protocols of how to set and adjust APRV based on TCAV have been discussed in detail elsewhere [93].

**Fig. 3:**
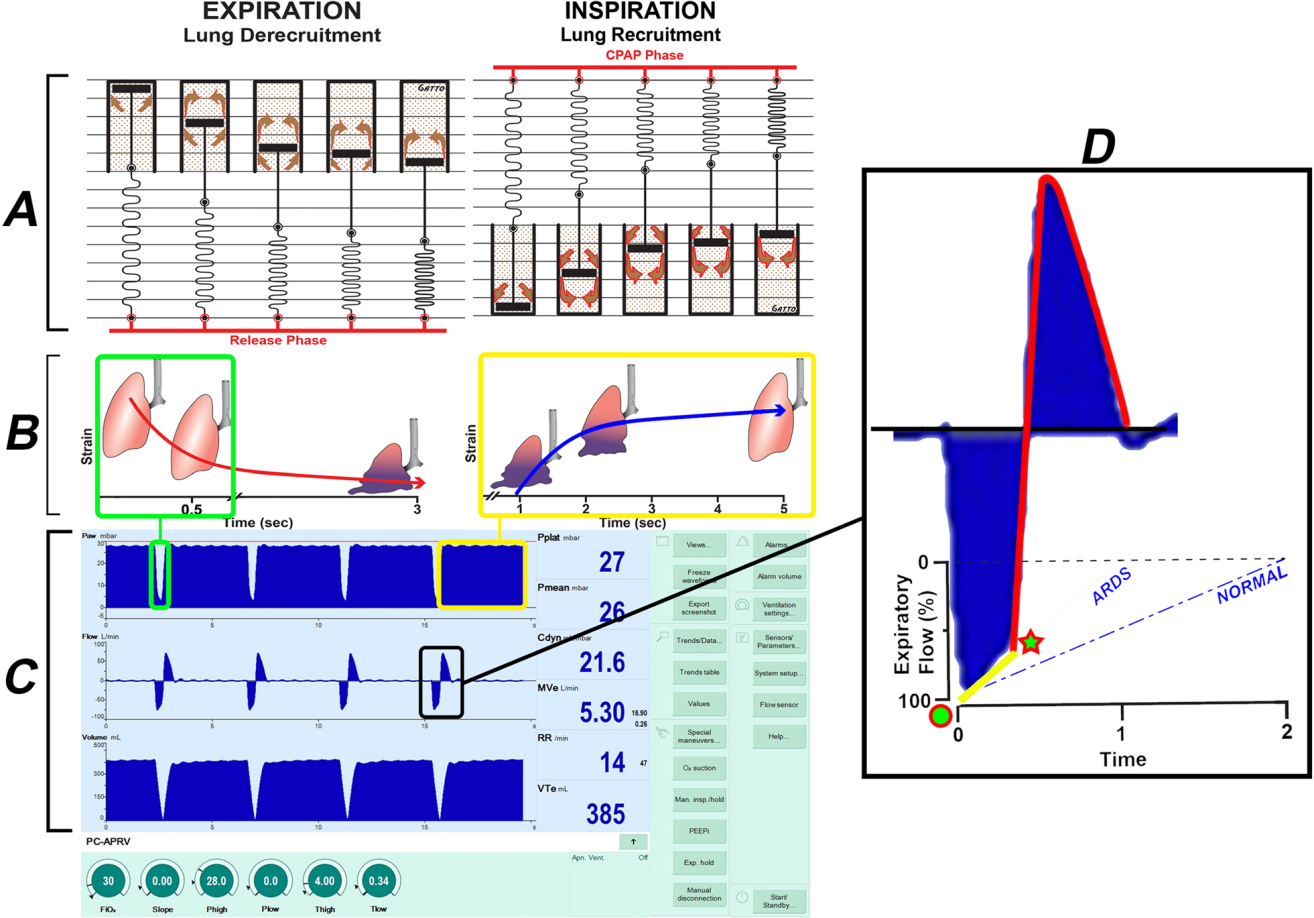
The ability of the TCAV method to stabilize and then open the lung is based on opening and collapse time constants, which are greatly altered if pulmonary surfactant is deactivated, and the viscoelastic system by which the lung changes volume. **A** Viscoelastic lung volume change. EXPIRATION (Lung Derecruitment): Viscoelastic volume change can be modeled using the spring connected in parallel with a dashpot. When airway pressure begins to fall during the Release Phase (Fig. 2B), there is a very short time delay before alveolar collapse begins, followed by rapid collapse (spring), and then a gradual, continual collapse over time (dash moving slowing through the pot). INSPIRATION (Lung Recruitment): When airway pressure is reapplied during the CPAP Phase (Fig. 2B, CPAP Phase), the reverse sequence of events occurs during lung opening: slight delay → rapid recruitment → gradual progressive recruitment. **B** Diagram of viscoelastic lung opening and collapse over time. If the expiratory time is very brief (≤ 0.5 s), lung tissue will not have time to collapse (green box). Lung tissue will continue to recruit without a change in airway pressure for as long as the CPAP Phase is applied. (yellow boxes). **C** A ventilator monitor showing typical TCAV method Pressure/ Flow/ Volume/ Time curves. Using the TCAV method the extended CPAP Phase continually 'nudges' the lung open over time (yellow boxes) (Additional file 3, Additional file 4) and establishes durable lung recruitment by not giving the lung sufficient time to collapse during the brief Release Phase (green boxes). **D** A blow-up of the expiratory and inspiratory flow curves is seen on the ventilator monitor (black box). The animal being ventilated had ARDS so the slope of the expiratory flow curve (Slope_EF_) was very steep (*ARDS*, yellow line) as compared to the Slope_EF_ in a healthy lung (*NORMA*L, blue dashed line). At the termination of the brief Release Phase (green star) the lung is rapidly reinflated to the set CPAP Pressure. Panel A Adapted from Reference [29], under terms of the Creative Commons Attribution 4.0 International License

*Using time to prevent alveolar collapse*: The most critical aspect of the TCAV method is setting the T_Low_ correctly, thus we focus most of our efforts on explaining the physiology of T_Low_. The brief Release Phase effectively controls the duration of passive exhalation flow, thereby regulating and retaining lung volume (EELV) and exhaled lung volume (V_T_). Fundamentally, precise control of integrals of volume (i.e., flow and time) permit direct control of lung volume change between inspiration and expiration. More traditional approaches with PEEP exhales to a pressure, indirectly controlling lung volume change. The retained volume with TCAV secondarily produces a TC-PEEP, even though the P_Low_ is set at 0 cmH_2_O (Fig. 2B, TC-PEEP red dotted line) as EELV is controlled by time and not pressure where the lung simply does not have sufficient time to fully depressurize. Because lung strain is viscoelastic, which is modeled as a Spring & Dashpot (Fig. 3A) this suggests time is a controller of lung inflation, both increasing and decreasing in lung volume. Sequential time-dependent RACE should first be neutralized, creating the opportunity for gradual lung reopening that is enduring. The duration towards lung volume normalization takes hours to days and yields to the time dictate of lung micromechanics. Current methods of lung recruitment attempt to open the majority of the collapsed lung in seconds and minutes. Recruiting a large volume of collapsed lung quickly is problematic since a high level of PEEP would be necessary to prevent the re-collapse of this newly opened tissue with dysfunctional surfactant.

P_High_ is set to maintain lung inflation along the steep portion of the pressure–volume curve between functional residual capacity and total lung capacity. In practice, it is important to determine the intravascular volume status of a patient and if they are pre-load dependent. In a pre-load dependent state validated by an assessment such as passive leg raise maneuver, a fluid bolus has a favorable hemodynamic outcome without causing pulmonary edema [94]. Setting P-high from a volume control (VC) mode should equal Pplat, from a pressure control (PC) or dual control mode mode should equal Ppeak, and from HFOV should equal the Paw plus 2–4 cm H_2_O. If setting APRV with the TCAV method as the initial mode of mechanical ventilation, consider using the PO_2_ from arterial blood gas values where mild ARDS would set P_High_ 20–24 cm H_2_O, moderate 25–29 cm H_2_O, severe 26–30 cm H_2_O, and in some patients with obesity and heavy chest wall weight, P_High_ may need to be higher. Assessment of lung volume includes using the chest radiograph and looking at the curvature of the diaphragm. The ideal location for adequate lung volumes is a dome-shaped diaphragm that is located at the mid-clavicular line. Pressures may require adjusted as the clinical course changes, for either better (decreasing P_High_) or worse (increasing P_High_).

*Using time to gradually reopen alveoli*: During TCAV, the lung thus spends most of its time exposed to the CPAP Phase where the P_High_ must be high enough to induce gradual recruitment but not so high or abrupt as to cause volutrauma (Fig. 3B, C Inspiration, yellow box). The duration of T_Low_, on the other hand, cannot be longer than the shortest closure delay so that lung units do not have time to close before the next application of P_High_ begins (Fig. 3B, C Expiration, green box). Since recruitment of closed lung units depends on time as well as pressure, the longer the high pressure or CPAP Phase is applied, the more lung tissue that can be recruited (Fig. 3, Inspiration). The combination of this extended CPAP Phase and a rapid reinflation (Fig. 3D, red line) following a very brief Release Phase (Fig. 3D, star) functions as an inflate and brake ‘ratchet’ to gradually open the lung while simultaneously preventing re-collapse during expiration [95].

The T_Low_ (Release Phase) is the most critical component of the TCAV method. If the T_Low_ is set too long, set to target a V_T_ or intrinsic PEEP or to control PCO_2_ [even a fraction of a second], progressive atelectrauma results and offsets any gains produced during the CPAP Phase [19, 93, 96–98]. Furthermore, the optimal T_Low_ for a given patient is personalized and varies with factors such as the size of the artificial airway (i.e. endotracheal tube) and severity of lung injury. Since lung recoil forces are proportional to elastance of the respiratory system (E_RS_) and are high in severe ARDS, more rapid, passive exhalation (Fig. 3D, *ARDS* yellow line) occurs where the T_Low_ must be tuned and is briefer than in less severe lung disease. As the patient progresses toward recovery, the rate of lung emptying during expiration decreases, in which case the T_Low_ can be commensurately increased (Fig. 3D, *Normal* blue dashed line) [29].

**Fig. 4:**
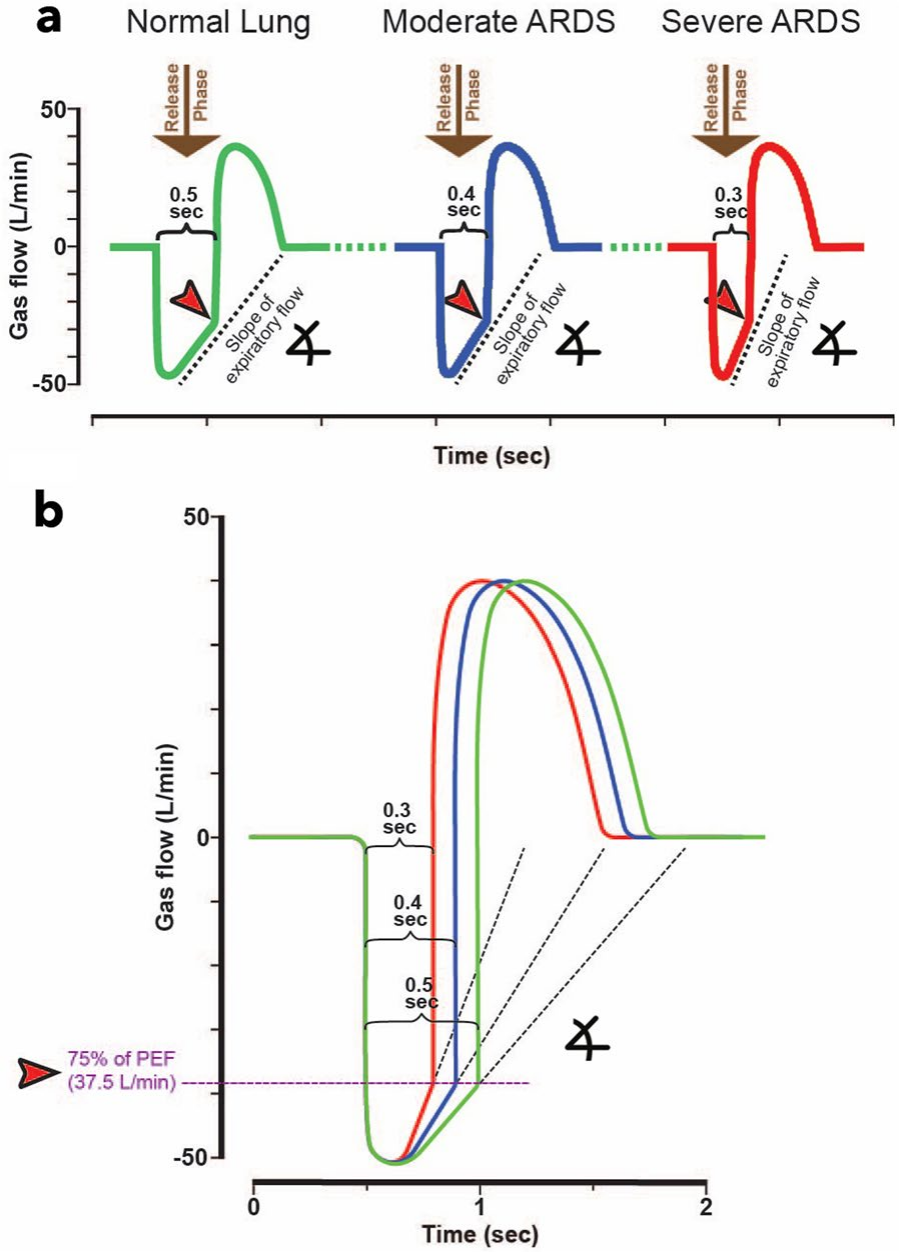
Expiratory Gas Flow/Time curve using the TCAV method to set T_Low_, the duration of the Release Phase (Fig. 2B, Release Phase). As lung injury increases from Normal Lung to Moderate and Severe ARDS, the respiratory system compliance (C_RS_) decreases, increasing the collapse recoil of the lung. The increased lung recoil causes faster gas flow during expiration resulting in a steeper slope of the expiratory flow curve (Slope_EF_). **a** Using the Slope_EF_ to set the Release Phase duration (Fig. 2B, Release Phase), the Normal Lung has a release time of 0.5 s, Moderate ARDS 0.4 s, and Severe ARDS 0.3 s. Expiratory flow is terminated (red arrowhead) by the clinician by adjusting the T_Low_, following which the lung is rapidly reinflated to the CPAP Phase (Fig. 2B, CPAP Phase). Thus, using the TCAV method personalizes and adapts the Release Phase (T_Low_) according to the patient’s lung physiology. **b** Calculation of the termination point on the expiratory flow curve using the TCAV method. Termination of expiratory flow (TEF) is calculated as 75% of the peak expiratory flow (PEF) (PEF 50L/min × 0.75 = TEF 37.5L/min). Adapted from Reference [104], under terms of the Creative Commons Attribution 4.0 International License

The explicit titration of T_Low_ in TCAV leverages time constants of expiration reflected in the mean slope of the expiratory flow (SLOPE_EF_) curve, which provides a breath-by-breath reflection of C_RS_ (Fig. 3D). Specifically, expiration is terminated when the magnitude of expiratory flow has decelerated to 75% of the peak expiratory flow (PEF) at the start of expiration. For example, if the PEF is 100 L/min multiplied by 0.75, the termination of PEFwould be the corresponding time (T_Low_) to achieve 75 L/min (Fig. 3D, star). A greater SLOPE_EF_ indicates decreased C_RS_, and terminating at 75% of PEF is achieved earlier in expiration (ie requires a T_Low_ reduction). Thus, T_Low_ can be tuned accordingly, resulting in a personalized method for setting ventilator parameters. Figure 4 illustrates how T_Low_ decreases as the SLOPE_EF_ increases going from *Normal lung* to *Severe ARDS* using the same calculation of termination at 75% of the PEF rate. (Fig. 4A, B: Expiratory time = 0.5 → 0.4 → 0.3secs). Importantly, adherence to this strategy is crucial for individualized lung protection, otherwise increasing the risk of severe VILI. The TCAV method has been shown to be lung protective when the T_Low_ is set to 75%, controlling EELV. For example, we have shown that while setting the T_Low_ to terminate at 75% of the PEF is highly protective and extending the T_Low_ with termination at 25% of PEF is extremely damaging in disorders of high E_RS_ [39]. Alternatively, because recoil forces are low in obstructive lung disease, setting the T_Low_ to terminate at 25% of PEF at the start of expiration is more suitable [99].

A recent viewpoint paper [100] suggests APRV is ill-advised and “should not routinely be used in patients with or at risk of acute respiratory distress syndrome outside of a clinical trial”, based on the analysis of eight randomized controlled trials. However, the data in question shows no evidence that APRV, when compared with conventional ventilation (i.e., VC or LV_T_ strategy), results in significant harm. Accordingly, there is no statistically justifiable conclusion on the inferiority of APRV, and any proclamation against the routine use of APRV based on current evidence is not supported; rather, more personalized and clinically efficacious modes of mechanical ventilation are needed [6]. Further, a notable challenge impeding the accurate scientific assessment of APRV lies in the widespread prevalence of myths and misconceptions consistently featured in scholarly publications [101]. Thus, any declaration that only the APRV mode is ill-advised for routine clinical use is inappropriate and may result in the loss of a powerful therapeutic tool in the arsenal of the clinician who must manage ventilation in patients with ARDS.

## Summary and conclusions

**Fig. 5:**
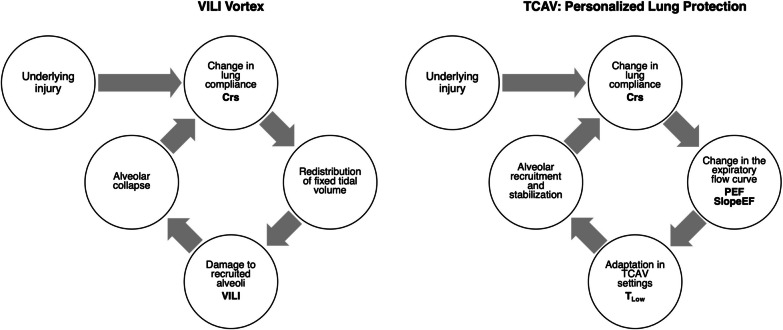
Closed-loop systems for both the *VILI Vortex* and *TCAV* personalized lung protection (Fig. 1). *VILI Vortex*—injury collapses lung tissue and reduces respiratory system compliance (C_RS_) → redistributing a fixed tidal volume into a heterogeneously damaged lung → leads to maldistribution of gas within the lung damaging alveoli by both atelectrauma and volutrauma → causing progressive lung collapse (VILI Vortex) → further reducing C_RS_. *TCAV*—injury collapses lung tissue and reduces C_RS_ → changes in C_RS_ are manifest as a change in the slope of the expiratory flow curve (Slope_EF_) → the Slope_EF_ is used to set the duration of the Release Phase and is thus directed by changes in the patient’s C_RS_ (Fig. 2B, Release Phase) → directed by changes in the patient’s C_RS_ the Release Phase is set sufficiently short to prevent alveolar collapse resulting in a gradual lung recruitment → lung recruitment increases C_RS_. The slope of the expiratory flow curve (Slope_EF_) can be used as a *dynamic feedback signal* to adaptively change the expiratory duration necessary to maintain lung stability. Changes in the Slope_EF_ will identify if C_RS_ is low or high and used to personalize and adaptively adjust the Expiratory Duration (T_Low_) necessary to maintain an open and stable lung, regardless of lung injury severity. The left side of the figure does not have this feedback mechanism which may lead to further alveolar collapse. On the right side of the figure the change in SlopeEF allows a stop and brake and adjustments made to T-low to halt the VILI Vortex

The mortality associated with ARDS remains high, despite the advent of lung protective ventilation strategies over twenty years ago. Seemingly well-founded strategies, such as LV_T_, HFOV, or OLA, despite their appeal, have not substantially reduced mortality. An analysis of the physiologic mechanisms behind VILI suggests these methods fail to eliminate its two principal causes: (1) generation of volutrauma at interfaces between collapsed and open parenchyma; and (2) avoidance of RACE. We propose these strategies do not account for the individual time dependence of opening and closing, which can vary across patients and subtypes of ARDS. Thus, a closed-looped personalized approach to lung protection can be achieved through a combination of the slow progressive reopening of collapsed lung tissue coupled with avoidance of closure using sufficiently brief, but patient-specific, expiratory durations in a closed-loop fashion (Fig. 5). TCAV is one approach to achieving these goals in a manner that adapts to the changing pathophysiology and clinical requirements of an individual patient using the Slope_EF_ as a dynamic, bedside tool. There may be other viable variations on this theme, but TCAV provides proof of concept through its demonstrated efficacy in patients and animal models [79, 81, 87, 88, 96, 102, 103], particularly when applied preemptively to the lung at risk for VILI. It may be time to consider altering the standard of care in ARDS to ventilation strategies that exploit such a personalized approach.

### Supplementary Information

**Additional file 1: Video S1.** Interdependent alveoli Hexagon model with a central area of instability. Acute Respiratory Distress Syndrome (ARDS) causes alveolar instability secondary to capillary leak and surfactant deactivation. Atelectrauma occurs due to the excessive ‘peeling’ stress as the alveolar wall in apposition peels apart. Collapsed alveoli act as a stress multiplier, causing overdistension and excessive strain on the walls of adjacent alveoli [doi.org/https://doi.org/10.1152/japplphysiol.00123.2017].

**Additional file 2: Video S2.** Interdependent alveoli model with a central area of collapse acting as a stress multiplier. With each breath, there is an excessive dynamic strain on the normal alveolar walls surrounding the collapsed or edema-filled tissue. [doi.org/https://doi.org/10.1152/japplphysiol.00123.2017].

**Additional file 3: Video S3.** Subpleural alveoli (spherical objects with bright borders) in a rat Acute Respiratory Distress Syndrome (ARDS) model. Extensive atelectasis (consolidated red areas) is present at atmospheric pressure. Gradual alveolar recruitment occurs incrementally following an applied airway pressure over an extended inspiratory time. [doi.org/https://doi.org/10.1152/japplphysiol.90735.2008].

**Additional file 4: Video S4.** Excised rat lung Acute Respiratory Distress Syndrome (ARDS) model. The pink area is inflated tissue, and darker red areas are atelectatic. Lung tissue recruits incrementally following an applied airway pressure over an extended inspiratory time. [doi.org/https://doi.org/10.1152/japplphysiol.90735.2008].

## Data Availability

Not applicable.
